# *Methylobacterium* infection of an arthritic knee

**DOI:** 10.1099/jmmcr.0.005173

**Published:** 2019-01-29

**Authors:** Eric T. Beck, Allen C. Bateman, Darin Maccoux

**Affiliations:** ^1^​ ACL Laboratories, West Allis, WI 53227, USA; ^2^​ Aurora Health Care, Milwaukee, WI 53215, USA; ^3^​ Wisconsin State Laboratory of Hygiene, Madison, WI 53718, USA

**Keywords:** osteoarthritis, *Methylobacterium*, Synvisc, Hylan G-F 20

## Abstract

**Introduction:**

Osteoarthritis (OA) is a common cause of knee pain in older adults. OA is primarily caused by deterioration of cartilage in the knee, which decreases the ability of synovial fluid to absorb shock and increases the opportunity for bones of the joint to rub together. Hylan G-F 20 (Synvisc-One) is a compound that can be injected directly into the knee to help combat the pain associated with OA by lubricating and cushioning the joint.

**Case presentation.:**

A 92-year-old male reported to his primary care provider with complaints of pain due to OA. An ultrasound-guided injection of Hylan G-F 20 was administered without complication; however, the patient presented to an emergency department approximately 10 h after the injection complaining of stabbing pain and swelling in the same knee. Specimens submitted for culture 12 h post-injection yielded a *Methylobacterium* spp. that was identified following biochemical testing, MALDI-TOF (matrix-assisted laser desorption/ionization-time of flight) MS analysis and bacterial sequencing. Interestingly, symptoms began to subside following aspiration of synovial fluid, and new cultures of synovial fluid collected 24 h post-Hylan G-F 20 injection were negative for the presence of *Methylobacterium*. The patient’s knee returned to baseline with diminished pain due to OA approximately 1 week after the initial injection without antibiotic treatment.

**Conclusion:**

We report short-term complications following treatment of OA with a *Methylobacterium*-contaminated lot of Hylan G-F 20.

## Introduction

Osteoarthritis (OA) affects more than 25 % of the adult population in the USA; it is most commonly associated with the elderly, but traumatic injury at any age can also be a cause [1]. OA is the most common degenerative joint disease and is characterized by progressive loss and destruction of articular cartilage, thickening of the subchondral bone, formation of osteophytes, inflammation of the synovium, degeneration of knee ligaments and hypertrophy of the knee capsule [2]. The primary symptoms of OA include chronic pain, joint instability and stiffness [3].

Treatment for OA consists of approaches to minimize the symptoms and includes: lifestyle changes (more exercise or changes in diet), pain relievers, physical or occupational therapy, steroid injections, viscosupplement injections and arthroplasty. Hylan G-F 20 (Synvisc-One; Sanofi Genzyme) is a viscosupplement gel mixture of hyaluronan. Significant quantities of hyaluronan naturally occur in the human joints, and act as lubricant and a shock absorber so the joint functions appropriately. The medication is injected directly into the patient’s knee to supplement the natural hyaluronan and is indicated for patients with OA who have not had sufficient pain relief from lifestyle changes, pain relievers or physical therapy. Side effects associated with Hylan G-F 20 injection include: pain, swelling, heat, redness, or fluid build-up in or around the knee. Hylan G-F 20 can help reduce OA symptoms for up to 6 months [4]. We report here a unique infection following injection of Hylan G-F 20 in a 92-year-old patient with OA.

## Case report

A 92-year-old male reported to his primary care provider with complaints of pain due to OA in his left knee. The patient received an ultrasound-guided injection containing 48 mg Hylan G-F 20 (Synvisc-One; Sanofi Genzyme) without complication. Approximately 8 h following the procedure, the patient reported stabbing pain and swelling in his left knee and ultimately presented to an emergency department 10 h post-injection. The patient’s vital signs were normal and outside of pain, the only other symptom was a decreased range of motion in his knee (limited to 90–120° of movement) compared to a full range of motion prior to the injection and effusion. Following discussion between the primary care provider who performed the injection and the emergency department provider, the patient’s joint was aspirated and nearly 100 ml straw-coloured synovial fluid was removed and sent to a microbiology laboratory for culture. Following aspiration, the patient reported improvement and was able to leave the emergency room without assistance. The patient returned to his primary care provider the following morning for follow-up, and while the knee remained swollen, the complaints of pain had decreased. The primary care provider collected an additional synovial fluid specimen for culture and submitted it to the microbiology laboratory. Seven days after the initial ultrasound-guided injection, the patient was seen by his primary care provider for further follow-up. At this time the symptoms were nearly completely dissipated and the patient’s range of motion had returned to baseline without undergoing additional treatment. In addition, the complaints due to OA had also subsided, indicating that aside from the initial pain the medication performed as expected.

## Diagnosis

**Fig. 1.**
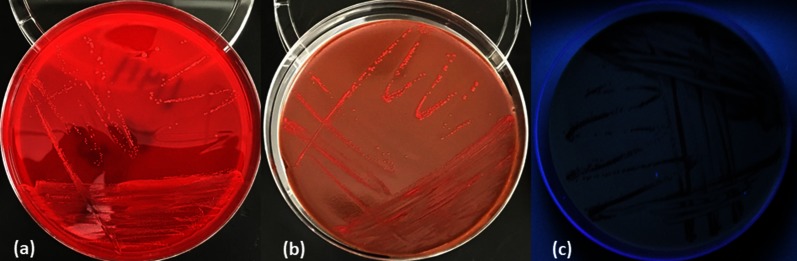
Subculture of the *Methylobacterium* on agar after 5 days of incubation; (a) 5 % sheep blood agar, (b) chocolate agar, (c) 5 % sheep blood agar under UV light.

**Fig. 2.**
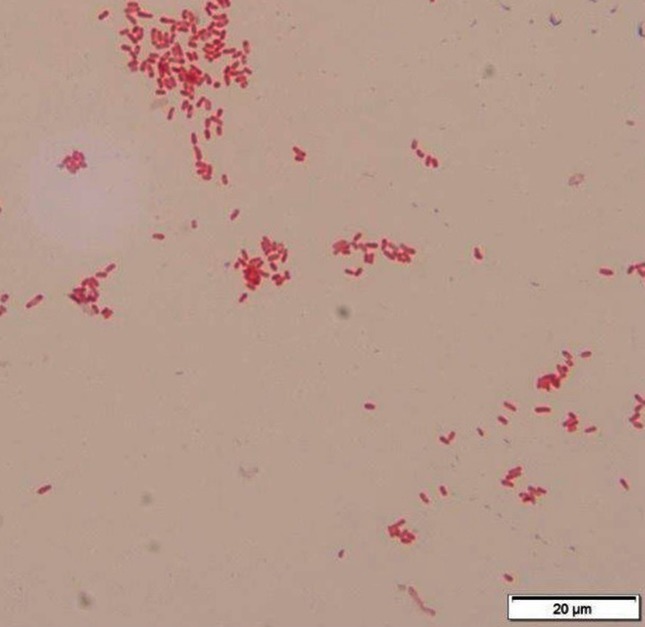
Gram stain of the *Methylobacterium.*

In the microbiology laboratory, nearly 96 h after initial sample collection, culture of the first synovial fluid specimen (collected approximately 12 h following Hylan G-F 20 injection) revealed the presence of a small pink, mucoid colony that did not grow on MacConkey agar (Fig. 1a, b). The organism was a catalase positive, weakly oxidase positive, vacuolated, Gram-negative bacillus (Fig. 2). In addition, the colonies absorbed UV light and appeared black under a Wood’s Lamp (Fig. 1c). Based on these characteristics, the organism was presumptively identified as a *Methylobacterium* species, but it failed to yield an identification on a Vitek MS MALDI-TOF instrument (bioMérieux). Of note, the synovial fluid culture of the specimen collected 24 h post-Hylan G-F 20 injection was ultimately finalized as negative 5 days post-inoculation. At this point, nearly a week after the initial Hylan G-F 20 injection, the patient’s symptoms had almost completely resolved and his range of motion had returned to baseline without antibiotic treatment. *Methylobacterium* spp. are generally considered environmental organisms; however, they have been associated with hospital-acquired infections, in particular relation to endoscope reprocessing, and have occasionally been reported as a cause of sterile site infections, including synovitis [5, 6]. The initial presentation in this case was indicative of infection; however, the inability to isolate the organism from the specimen collected 24 h post-injection and spontaneous resolution of the infection without antibiotic treatment contradicted this conclusion.

In an effort to confirm the identity of the organism, in the event that the patient’s symptoms returned, the isolate was submitted to the Wisconsin State Laboratory of Hygiene (WSLH), USA, for additional testing. At this time, a posting to the American Society for Microbiology’s ClinMicroNet ListServ (this is a closed ListServ discussion group for doctoral-level diagnostic microbiology laboratory directors that requires application through the American Society of Microbiology’s website) [7] notified members of a recent recall letter from Sanofi Genzyme that reported a specific lot of its Synvisc-One product was being recalled due to contamination with *Methylobacterium thiocyanatum*. A review of the patient’s record indicated that the contaminated lot reported by Sanofi Genzyme was indeed injected into this patient’s knee. The rapid onset of pain and ability to isolate the organism at 12 h but not 24 h post-injection indicates a transient bacterial presence due to the injection and not a true infection. The fact that the symptoms resolved without further antibiotic treatment seem to confirm this finding.

At the WSLH, additional testing on a Bruker Biotyper MALDI-TOF instrument (Bruker-Daltonics) utilizing the Bruker RUO database (Bruker-Daltonics) and the MicrobeNet database (Centers for Disease Control and Prevention) [8], confirmed the identity of a *Methylobacterium* spp., but could not identify the isolate to the species level. Approximately the first 500 bp of the 16S ribosomal RNA gene of the isolate were sequenced and compared to the National Center for Biotechnology Information (NCBI) sequence database sponsored by the United States National Institutes of Health (NIH) using the Basic Local Alignment Search Tool (blast) [https://blast.ncbi.nlm.nih.gov/Blast.cgi (accessed 23 March 18)]. The results showed greater than 99 % identity to both *M. thiocyanatum* and *Methylobacterium populi.* Because the isolate again could not be identified definitively to the species level, it was ultimately identified as *Methylobacterium* spp. most closely related to *M. populi* and *M. thiocyanatum*.

## Outcome and follow-up

Treatment consisted solely of synovial fluid aspiration 12 h post-Hylan G-F 20 injection. Following aspiration, the patient’s symptoms resolved spontaneously over the next week. The patient’s OA pain also dissipated as expected following injection of Hylan G-F 20. The range of motion returned to baseline and no lingering effects were observed at a 6 week follow-up visit.

## Discussion

*Methylobacterium* spp. are common environmental organisms. In rare instances, these bacteria have been associated with human infections of the following: blood, bone marrow, sputum, pleural effusion, peritoneal fluid, cerebrospinal fluid, synovium and skin [5, 9–16]. These infections are often associated with immunocompromised patients. We report here a case of *Methylobacterium* spp. isolation from the synovial fluid of an otherwise healthy 92-year-old male following injection of the OA treatment medication Hylan G-F 20.

While the isolation of this organism from a synovial fluid specimen is not unique, the inability to isolate the bacteria from the same source 12 h later followed by spontaneous resolution of the symptoms without additional treatment is. This finding left both the provider and the microbiology laboratory with a diagnostic conundrum. On one hand, the patient presented with signs and symptoms of an infection following an injection to the site of pain and isolation of an organism that has been associated with infections. On the other hand, the signs and symptoms of infection started approximately 8 h after injection, which is inconsistent with infection of such a slow-growing organism like *Methylobacterium* spp. In addition, the inability to subsequently isolate the organism 24 h after the initial injection and spontaneous resolution of symptoms made it difficult to determine whether the isolation of *Methylobacterium* spp. represented a clinically relevant finding. Unfortunately, tests to identify inflammatory markers (e.g. C-reactive protein) were not performed, which may have helped better understand whether this represented a clinically relevant infection in a more timely manner.

Fortunately, informational updates shared on the ClinMicroNet group allowed for rapid identification of the bacterial source, and allowed the laboratory and the provider to determine the best course of action for the patient [7]. This example underscores the importance of such ListServ groups in the laboratory profession. Based on this information, the Wisconsin Department of Public Health and the WSLH were able to quickly notify local infection control practitioners and clinical microbiology laboratories to the possibility of infection due to contaminated Synvisc-One. In addition, the United States Centers for Disease Control and Prevention was consulted to determine whether additional infection control steps were required.

In conclusion, we have reported the isolation of a *Methylobacterium* spp. organism from the synovial fluid of a patient who recently received an injection of Hylan G-F 20. Unrelated and concurrent to this event, the manufacturer of the Hylan G-F 20 issued a recall notice for this specific lot of Hylan G-F 20 due to contamination with *M. thiocyanatum*. As far as the authors are aware, this is the first and only report of isolation of a *Methylobacterium* from a patient following injection with contaminated Synvisc-One.
